# Relationship of Transmural Variations in Myofiber Contractility to Left Ventricular Ejection Fraction: Implications for Modeling Heart Failure Phenotype With Preserved Ejection Fraction

**DOI:** 10.3389/fphys.2018.01003

**Published:** 2018-08-24

**Authors:** Yaghoub Dabiri, Kevin L. Sack, Semion Shaul, Partho P. Sengupta, Julius M. Guccione

**Affiliations:** ^1^Department of Surgery, University of California, San Francisco, San Francisco, CA, United States; ^2^Section of Cardiology, West Virginia University Heart and Vascular Institute, West Virginia University, Morgantown, WV, United States

**Keywords:** heart failure and preserved ejection fraction, left ventricle, myocardial contractility, finite element method, simulation

## Abstract

The pathophysiological mechanisms underlying preserved left ventricular (LV) ejection fraction (EF) in patients with heart failure and preserved ejection fraction (HFpEF) remain incompletely understood. We hypothesized that transmural variations in myofiber contractility with existence of subendocardial dysfunction and compensatory increased subepicardial contractility may underlie preservation of LVEF in patients with HFpEF. We quantified alterations in myocardial function in a mathematical model of the human LV that is based on the finite element method. The fiber-reinforced material formulation of the myocardium included passive and active properties. The passive material properties were determined such that the diastolic pressure-volume behavior of the LV was similar to that shown in published clinical studies of pressure-volume curves. To examine changes in active properties, we considered six scenarios: (1) normal properties throughout the LV wall; (2) decreased myocardial contractility in the subendocardium; (3) increased myocardial contractility in the subepicardium; (4) myocardial contractility decreased equally in all layers, (5) myocardial contractility decreased in the midmyocardium and subepicardium, (6) myocardial contractility decreased in the subepicardium. Our results indicate that decreased subendocardial contractility reduced LVEF from 53.2 to 40.5%. Increased contractility in the subepicardium recovered LVEF from 40.5 to 53.2%. Decreased contractility transmurally reduced LVEF and could not be recovered if subepicardial and midmyocardial contractility remained depressed. The computational results simulating the effects of transmural alterations in the ventricular tissue replicate the phenotypic patterns of LV dysfunction observed in clinical practice. In particular, data for LVEF, strain and displacement are consistent with previous clinical observations in patients with HFpEF, and substantiate the hypothesis that increased subepicardial contractility may compensate for subendocardial dysfunction and play a vital role in maintaining LVEF.

## Introduction

Heart Failure (HF) is the only cardiovascular disease for which incidence, prevalence, morbidity, mortality, and costs are not decreasing. According to the 2017 Update (Benjamin et al., 2017), the prevalence of HF has increased from 5.7 million (2009 to 2012) to 6.5 million (2011 to 2014) in Americans >20 years of age and projections show prevalence will increase 46% by 2030, resulting in over 8 million adults with HF (Heidenreich et al., 2013). In 2012, the total cost for HF was estimated to be $31 billion and projections show that by 2030, the total cost will increase to $70 billion or roughly ~$244 for every US adult (Heidenreich et al., 2013). Among patients hospitalized for an HF incident, 47% had HF with preserved ejection fraction (HFpEF) or systolic function, which is the focus of this paper.

The mechanism of the development of HFpEF is not well-understood (Aurigemma and Gaasch, 2004; Shah and Solomon, 2012; Steinberg et al., 2012; Sengupta and Marwick, 2018), and optimal treatment options remain unclear (Vasan et al., 1995; Bhuiyan and Maurer, 2011). Recent studies have suggested that HFpEF is associated with transmural changes in myocardial deformation (Shah and Solomon, 2012; Omar et al., 2016, 2017). Understanding the transmural variations in left ventricular (LV) mechanics associated with HFpEF may offer pathophysiological insights for developing potential therapeutic targets. We therefore explored a physics-based mathematical [finite element (FE)] model of the normal human LV to test the hypothesis that reduced subendocardial contractility combined with compensatory high subepicardial contractility may help in preserving LVEF independent of changes in myocardial geometry and material properties. We used our established computational framework in this paper. To the best of our knowledge, this is the first study that quantifies the development of HFpEF based on transmural variation in contractility, using patient-specific parameters.

## Methods

### Patient data

*In vivo* echocardiographic recordings were obtained under a protocol approved by our institutional review board. Individual patients provided informed consent and anonymized data were sent to a core laboratory for analysis.

### Geometry considerations

The ventricle model pertains to a normal human subject. The LV was modeled as a truncated thick-walled ellipsoid (Mercier et al., 1982; LeGrice et al., 2001). Based on echocardiography recordings for end diastolic volume (EDV), LV diameter and wall thicknesses for the posterior and septal wall, we back-calculated ellipsoidal surfaces for the endocardium and epicardium at end diastole (ED).

Using a linearly regressed estimation of the unloaded LV cavity volume V0 (Klotz et al., 2006) we scaled the dimensions of the endocardium surface to match the calculated volume V0. The epicardium dimensions were then scaled to maintain the same myocardial wall volume ascertained at the ED configuration (preservation of mass).

**Figure 1:**
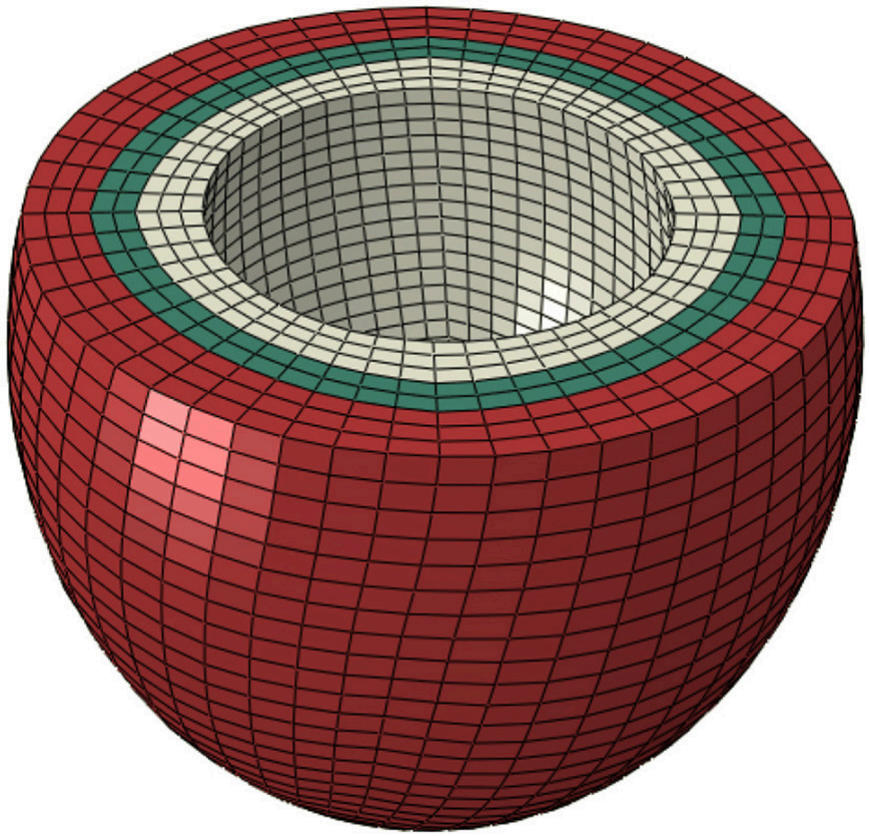
In normal conditions, contractility (T_max_) was uniform in all layers (scenario 1). To simulate no contraction in the subendocardial region, contractility in three layers in white was set to zero (scenario 2). The three layers in red were used to simulate alterations in subepicardial contractility (scenario 3). The three white layers, the two green layers, and the three red layers comprise subendocardial, midmyocardial, and subepicardial regions, respectively.

TruGrid (XYZ Scientific Applications Inc, Pleasant Hill, California, USA) was used to mesh LV surfaces. The ventricle was meshed to produce eight layers through the radial direction (Figure 1). Finite element calculations were performed in ABAQUS (SIMULIA, Providence, RI, USA). The FE meshes are shown in Figure 1.

We used a rule-based approach coded in MATLAB 2012b (The MathWorks, Inc., Natick, Massachusetts, United States) to assign myofiber orientations to the centroid of each element in the meshed LV geometry. The aggregated myofiber orientation was assumed to present with an angle of −60° from the local circumferential direction on the epicardium surface that varies linearly through the LV wall thickness to an angle of +60° on the endocardial surface. This assumption is well-established in LV modeling studies (Carrick et al., 2012; Lee et al., 2013, 2015; Genet et al., 2014), and based on histological studies (Streeter et al., 1969), and diffusion tensor MRI studies (Lombaert et al., 2011).

### Constitutive equation and material parameters

The material formulation of the LV tissue includes passive and active properties. The passive behavior of the tissue was described using the model introduced by Holzapfel and Ogden (Holzapfel and Ogden, 2009; Göktepe et al., 2011). Briefly, the strain energy function used to compute passive stresses is composed of deviatoric (Ψ_*dev*_) and volumetric (Ψ_*vol*_) parts as follows:

(1) $$\begin{array}{r} {\text{Ψ}}_{dev}=\frac{a}{2b}e^{b\left(l_{1}-3\right)}+\sum_{i=f,s}\frac{a_{i}}{2b_{i}}\left\{e^{b_{i}{\left(l_{4i}-1\right)}^{2}}-1\right\}\\ +\frac{a_{fs}}{2b_{fs}}\left\{e^{b_{fs}{\left(l_{8fs}\right)}^{2}}-1\right\}\\ {\text{Ψ}}_{vol}=\;\frac{1}{D}\left(\frac{J^{2}-1}{2}-\ln\left(J\right)\right) \end{array}$$

where *a* and *b* represent isotropic stiffness of the tissue, *a*_*f*_ and *b*_*f*_ represent tissue stiffness in the fiber direction, and *a*_*fs*_ and *b*_*fs*_ represent the stiffness resultant from connection between fiber and sheet directions; *l*_1_, *l*_4*i*_, and *l*_8*fs*_ are invariants, defined as follows:

$$\begin{array}{l} l_{1}:=tr\left(C\right)\\ l_{4i}:=C:\left(f_{0}⊗f_{0}\right)\\ l_{8fs}:=C:sym\left(f_{0}⊗s_{0}\right) \end{array}$$

where ***C*** is the right Cauchy-Green tensor, and ***f***_0_ and ***s***_0_ are vectors specifying the fiber and sheet directions, respectively. *J* is the deformation gradient invariant, and *D* is a multiple of the Bulk Modulus *K* (i.e., *D* = 2/*K*).

The material constants *a*, *a*_*i*_, and *a*_*fs*_ scale the strain-stress curve, whereas material constants *b*, *b*_*i*_, and *b*_*fs*_ determine the shape of the strain-stress curve. To determine these parameters we used the End Diastolic Pressure Volume (ED PV) curve as described by Klotz et al. who reported an analytical expression for the ED PV curve based on a single PV point that is applicable for multiple species, including humans (Klotz et al., 2006). The LV EDV of 53 ml was recorded using echocardiography and the LV EDP of 14.3 mmHg was approximated from echocardiography data using Nagueh's formula (Nagueh et al., 1997).

**Figure 2:**
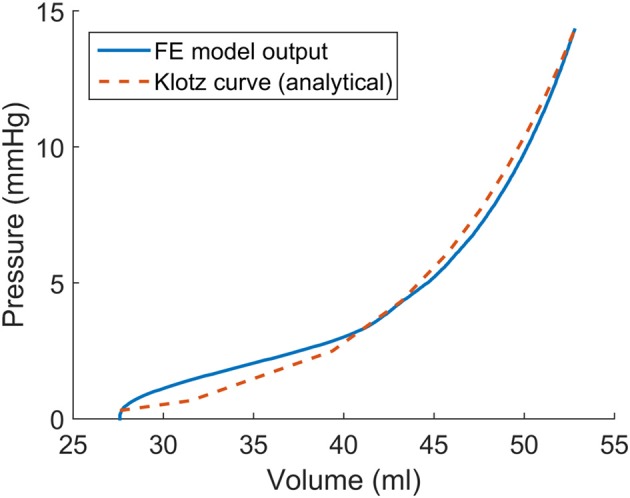
The passive material properties were determined such that the end diastolic pressure volume (ED PV) curve from finite element model was close to the experimental ED PV curve determined by Klotz et al. (2006).

The optimized material properties were found using an in-house Python script that minimized the error between the ED PV curve from the FE model and the analytical expression (Klotz et al., 2006). The sequential least squares (SLSQP) algorithm (Jones et al., 2001) was used in the Python script, and ABAQUS was used for the FE modeling, as the forward solver (Table 1 and Figure 2).

**Table 1:** Passive material properties that produced a pressure-volume curve close to the experimental pressure-volume curve (Figure 2).

***a* (MPa)**	***b***	***a*_*f*_ (MPa)**	***b*_*f*_**	***a*_*s*_ (MPa)**	***b*_*s*_**	***a*_*fs*_ (MPa)**	***b*_*fs*_**
6.832e^−4^	7.541	2.252e^−3^	14.471	3.127e^−4^	12.548	1.837e^−4^	3.088

The formulation for the active stress has been described extensively in the literature (Guccione and McCulloch, 1993; Walker et al., 2005; Genet et al., 2014; Sack et al., 2016). In short, the active stress in the myofiber direction was calculated as:

(2) $$\begin{array}{r} T_{0}=T_{max}\frac{Ca_{0}^{2}}{Ca_{0}^{2}+ECa_{50}^{2}}C_{t} \end{array}$$

where *T*_*max*_ is the isometric tension at the largest sarcomere length and highest calcium concentration, *Ca*_0_ is the peak intracellular calcium concentration, and

$$\begin{array}{l} C_{t}=\frac{1}{2}(1-cos\omega ),\\ \omega =\{\begin{array}{c} \pi \frac{t}{t_{0}}\;when\;0\leq t\leq t_{0}\\ \pi \frac{t-t_{0}+t_{r}}{t_{r}}\;when\;t_{0}\leq t\leq t_{0}+t_{r}{}^{\prime }\\ 0\;when\;t\geq t_{0}+t_{r} \end{array}\\ t_{r}=ml+b \end{array}$$

*m, b* = constants that govern the shape of the linear relaxation duration and sarcomere length relaxation.

Also,

$$\begin{array}{l} ECa_{50}=\frac{{\left(Ca_{0}\right)}_{max}}{\sqrt{\exp\left[B\left(l-l_{0}\right)\right]-1}},l=l_{R}\sqrt{2E_{ff}+1} \end{array}$$

where *E*_*ff*_ is the Lagrangian strain in the fiber direction, *B* is a constant that governs the shape of the peak isometric tension-sarcomere length relation, *l*_0_ is the sarcomere length that does not produce active stress, *l*_*R*_ is the sarcomere length with the stress-free condition, and (*C*_*a*_0_)*max*_ is the maximum peak intracellular calcium concentration.

The active stress was added to the passive stress to compute total stress:

(3) $$\begin{array}{r} S=S_{Passive}+T \end{array}$$

where ***S*** is the total stress.

The boundary and load conditions generally follow the ABAQUS Living Heart Model (Baillargeon et al., 2014, 2015; Sack et al., 2016). In particular, the center of the LV proximal cross-section (base) was fixed. The average rotation and translation of nodes of the endocardial annulus were coupled to the center of the LV base. This boundary condition prevents rigid body rotation, but allows inflations and contractions of the annulus. The nodes of the base were fixed in the longitudinal direction. A pressure load was applied to the LV surface to simulate diastole, whereas the contraction of the LV muscles caused systole. Surface-based fluid cavities and fluid exchanges were used to model blood flow (ABAQUS Analysis User's Guide).

When *T*_max_ is changed in Equation (2), the total contractile force of the tissue is altered, and other parameters related to the passive and active material formulations (Equations 1, 2) either do not change or change in a consistent way. We can prescribe different values of T_max_ in transmural layers to introduce regionally varying contractility throughout the LV. We considered six scenarios with different contractile properties, as explained in Table 2. Homogenous contractile properties were considered in scenario 1, which also served to establish a baseline value for normal T_max_. T_max_ was calibrated to produce the echocardiogram-recorded value for end- systolic volume (ESV) for this patient (24.8 ml). To simulate the diseased condition, subendocardial contractility was set to zero by setting T_max_ = 0 (scenario 2). To recover ESV, a scenario was considered in which T_max_ was increased in the subepicardial layers (scenario 3). To further assess the effects of transmural contractility, three more scenarios with different contractility in the transmural layers were created. In scenario 4, T_max_ in all regions was reduced by 50%. In scenario 5, T_max_ was set to zero in subepicardial and midmyocardial regions. In scenario 6, T_max_ was set to zero in the subepicardial region.

**Table 2:** Six scenarios were created to examine effects of contractility (T_max)_ on EF.

**Scenario**	**T**_**max**_ **(MPa)**			**EF (%)**	**ESV (ml)**	**ESP (mmHg)**	**Torsion (degrees)**	**Strain (%)**		
	**Three inner layers (subendocardium)**	**Two middle layers (midmyocardium)**	**Three outer layers (subepicardium)**					**E_l_**	**E_c_**	**E_r_**
1	0.086	0.086	0.086	53.2	24.7	88.9	24.7	−8.5	−29.7	44
2	0.0	0.086	0.086	40.5	31.4	94.5	26.7	−4.5	−15.8	25.3
3	0.0	0.086	0.117	53.2	24.7	92.8	30.2	−6.1	−28.1	39.9
4	0.043	0.043	0.043	13.3	45.8	82.6	18.6	−5.7	−0.6	12.9
5	0.086	0.0	0.0	0.3	52.6	63.2	−7.6	−7.6	2.7	4.9
6	0.086	0.086	0.0	12.7	46.0	80.1	−13.0	−5	−4.4	7.15

*Scenario 1 represents the normal condition; scenario 2 represents zero subendocardial contractility; scenario 3 represents zero subendocardial contractility and increased subepicardial contractility (an HFpEF condition); scenario 4 represents decreased contractility in all regions; scenario 5 represents zero midmyocardial and subepicardial contractility, and scenario 6 represents zero subepicardial contractility. For scenario 1, the computational EF matched the experimental EF. For all scenarios, EDV = 53 ml, EDP = 14.3 mmHg. El, Ec, and Er are, respectively, ES strain in longitudinal, circumferential and radial directions. The circumferential and radial strains were computed using the nodes located at the endocardial annulus (base of the LV)*.

To calculate LV torsion, we use the following formula (Aelen et al., 1997; Rüssel et al., 2009).

(4) $$\tau =\frac{\left(\emptyset_{\text{apex}}-\emptyset_{\text{base}})\times (\rho_{\text{apex}}+\rho_{\text{base}}\right)}{\text{2D}}$$

**Figure 3:**
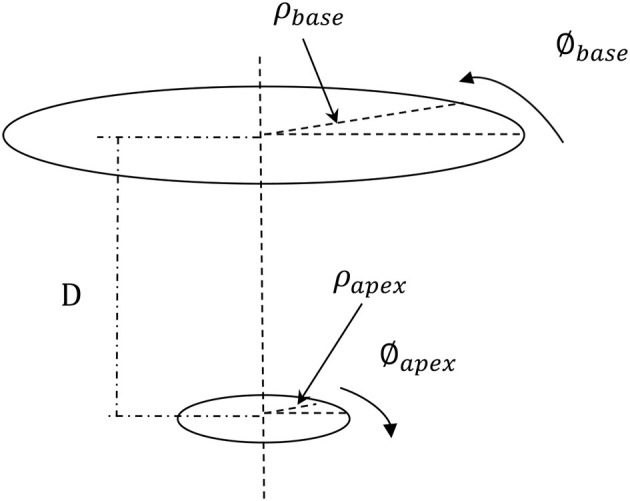
The torsion of the LV was computed based on the apical and basal rotations, the apical and basal radius, and the distance between the apex and base. The formula used to compute the LV torsion (Equation 4) makes the LV torsion comparable for hearts of different sizes (Aelen et al., 1997; Rüssel et al., 2009). The positive rotation is counterclockwise when seen from apex.

Where τ is normalized LV torsion; Ø_apex_ and Ø_base_ are rotations in the apex and base, respectively; ρ_apex_ and ρ_base_ are the radius of the apex and base, respectively; and *D* is the distance between the apex and base (Figure 3).

## Results

**Figure 4:**
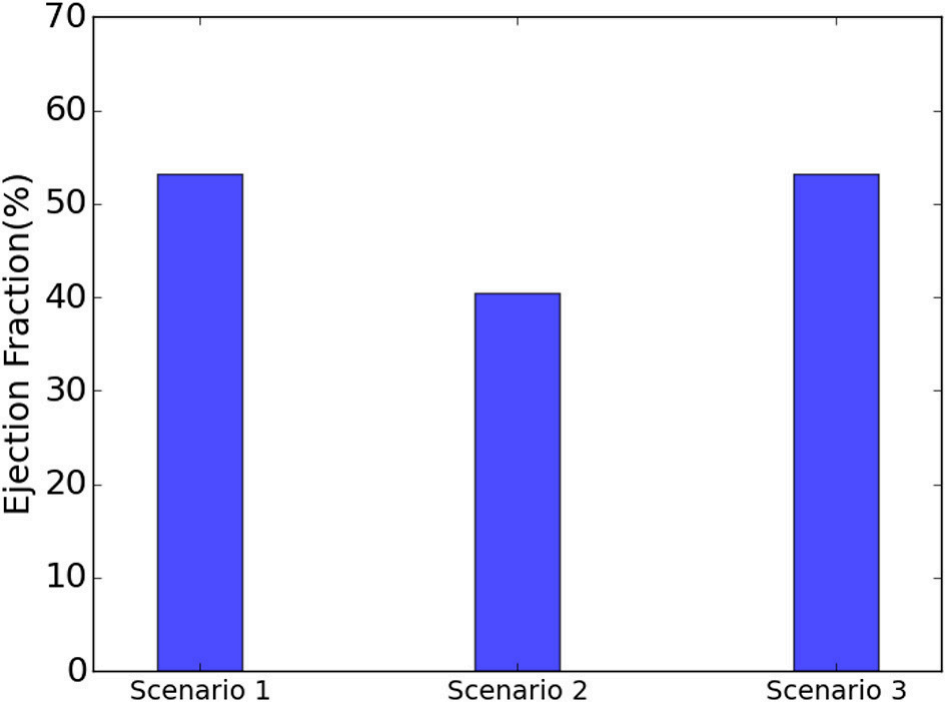
When the subendocardial contractility was zero, EF reduced by 23.9% relative to scenario 1 (scenarios 1 and 2). Increased subepicardial contractility recovered EF to scenario 1 (scenarios 1 and 3).

The EF decreased from 53.2 to 40.5% when T_max_ was set to zero in the subendocardial layers (Table 2 and Figure 4: scenario 2 vs. 1: 23.9% reduction in EF). The depressed contractility in the subendocardial region was enough to drop EF below 50%, producing HF with reduced EF (HFrEF). The EF normalized when T_max_ was increased in the subepicardial layers (Table 2 and Figure 4: scenario 3 vs. 1). This increased subepicardial contractility was enough to recover EF from the failing value of 40.5% and reach 53.2% (vs. 53.2% in the normal scenario). End-systolic pressure (ESP) and ESV increased when subendocardial contractility was zero. After subepicardial contractility increased, ESV and ESP decreased (Table 2, scenario 3 vs. 1 and 2).

The EF decreased by 75% when contractility decreased by 50% in all layers (Table 2: scenario 4 vs. 1). When subepicardial and midmyocardial contractility was zero, EF became almost zero (0.3% in scenario 5, Table 2). Similarly, when subepicardial contractility was zero, EF decreased dramatically compared to the normal scenario (12.7% in scenario 6 vs. 53.2% in scenario 1, Table 2). ESV noticeably increased and ESP decreased in scenarios 4, 5, and 6 vs. scenario 1.

When subendocardial contractility was zero, LV torsion increased (scenario 2 vs. 1). The torsion further increased after contractility in a remaining region was increased to compensate (scenario 3 vs. 1 and 2). The torsion decreased when contractility in all transmural regions decreased by 50% (scenario 4 vs. 1). The torsion reversed when midmyocardial and subepicardial contractility were decreased to zero (scenario 5 vs. 1). The reversed torsion increased when only subepicardial contractility was zero (scenario 6 vs. 5).

**Figure 5:**
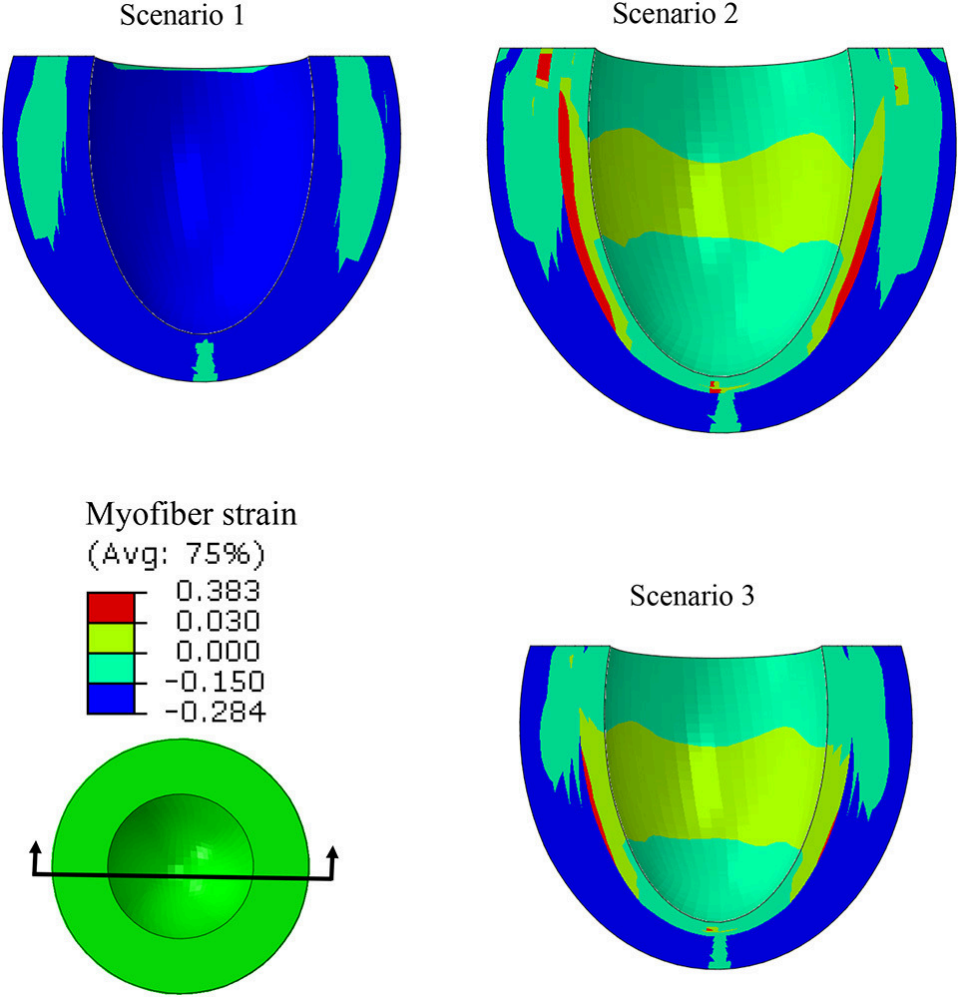
A long-axis view showing that at end systole, with uniform T_max_ (scenario 1), all layers experienced compressive strain in myofiber directions. When subendocardial contractility was zero, the strain pattern was altered (scenarios 1 and 2), but it partially recovered when subepicardial contractility increased (scenarios 1 and 3).

**Figure 6:**
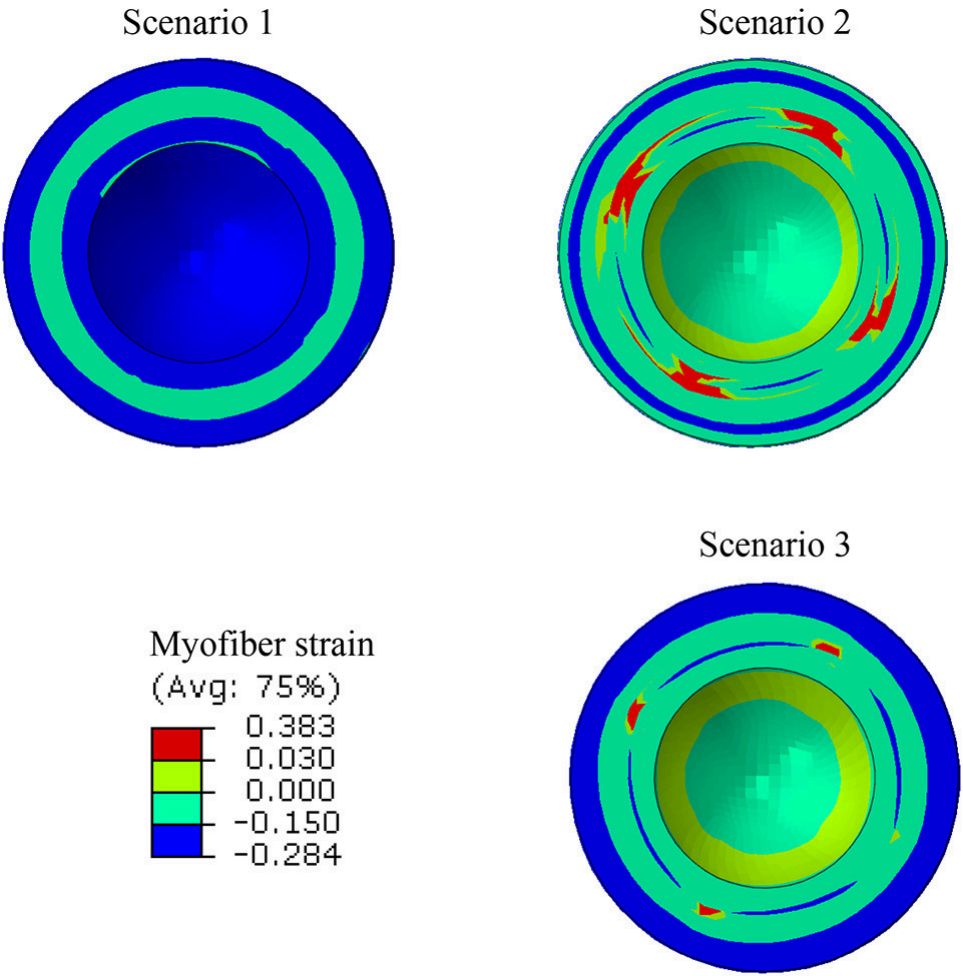
A short-axis view showing that the ES myofiber strain pattern altered when subendocardial contractility was zero (scenarios 1 and 2), but a partial recovery in strain pattern was observed when subepicardial contractility increased (scenarios 1 and 3).

**Figure 7:**
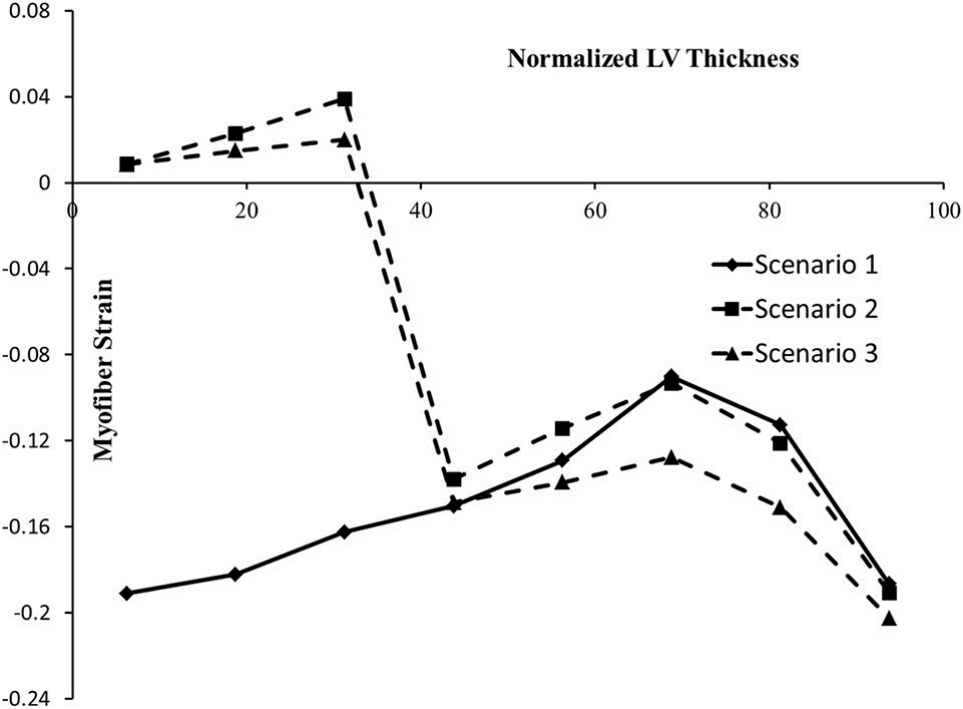
The ES myofiber strain at various points along LV thickness. In the horizontal axis, 0% represents the endocardium and100% represents the epicardium. The alterations in strains in scenario 2 are noticeable, compared to scenario 1. In scenario 3, the tensile strains decreased compared to scenario 2.

Strains (which are independent of displacement boundary conditions) were altered in diseased conditions. The global longitudinal, circumferential, and radial strains decreased in HFpEF, but recovered after subepicardial contractility increased (Table 2, scenarios 2 and 3 vs. scenario 1). In addition, the global strains decreased when contractility decreased by half in all layers, and when subepicardial and midmyocardial contractility were zero, and also when subepicardial contractility was zero (Table 2, scenarios 4, 5, and 6 vs. scenario 1). The direction of circumferential strain changed when midmyocardial and subepicardial contractility were both zero (Scenario 5 vs. 1, Table 2). With normal homogenous contractility (scenario 1), all layers experienced contractile strains (Figures 5–7). Regional changes in contractility to simulate HFrEF (scenario 2) and HFpEF (scenario 3) both presented with tensile strains in the subendocardial regions where contractility was set to zero (Figures 5–7). However, the increased subepicardial contractility in HFpEF had a global effect on strains throughout all layers, reducing the strains in all regions. Qualitatively, the transmural strain curve of the HFpEF case (scenario 3) replicated the pathological HFrEF curve (scenario 2), albeit with strains that were 23.8% lower on average.

**Figure 8:**
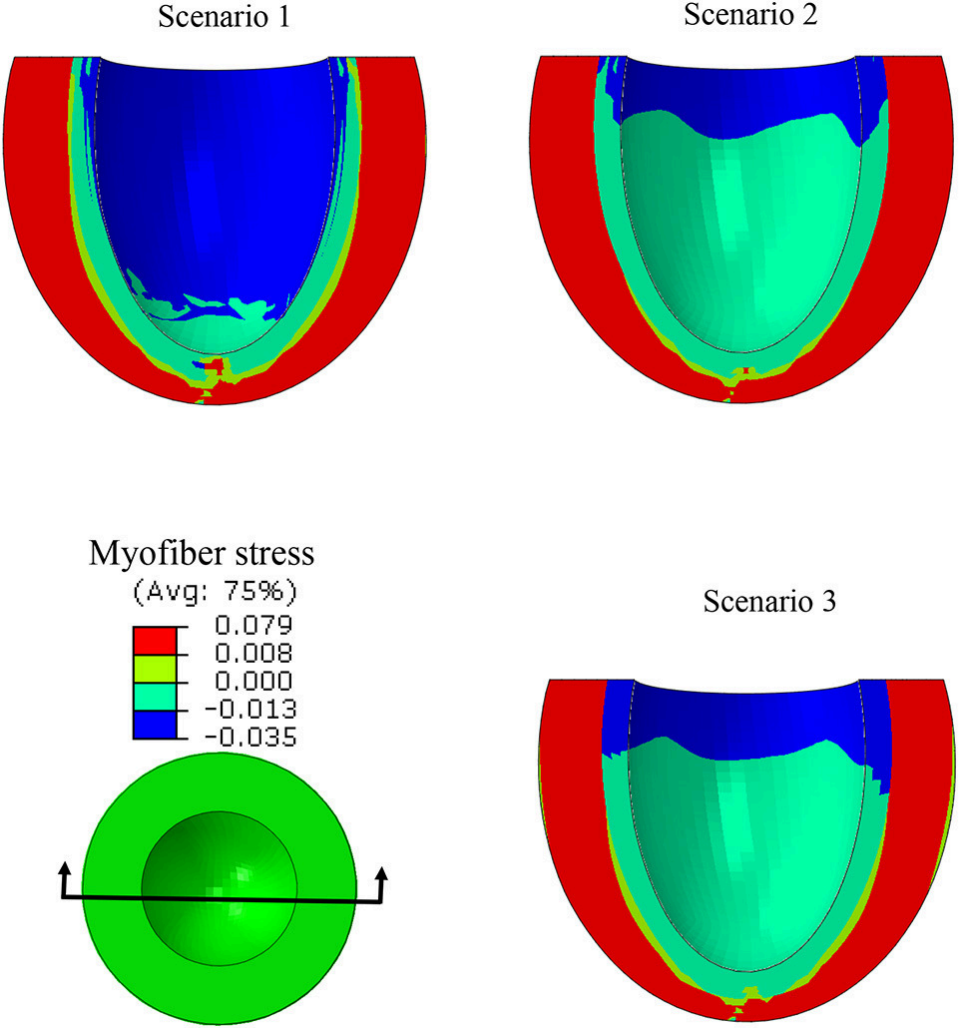
A long-axis view showing the ES myofiber compressive stress decreased when the subendocardial contractility was zero (scenarios 1 and 2). The stress pattern became partially similar to the normal case when subepicardial contractility increased (scenarios 1 and 3).

ES stress in the myofiber direction was noticeably reduced when subendocardial contractility decreased (scenarios 1 and 2, Figure 8). A trend to recovery in the stress distribution was observed when subepicardial contractility increased (scenarios 1 and 3, Figure 8).

**Figure 9:**
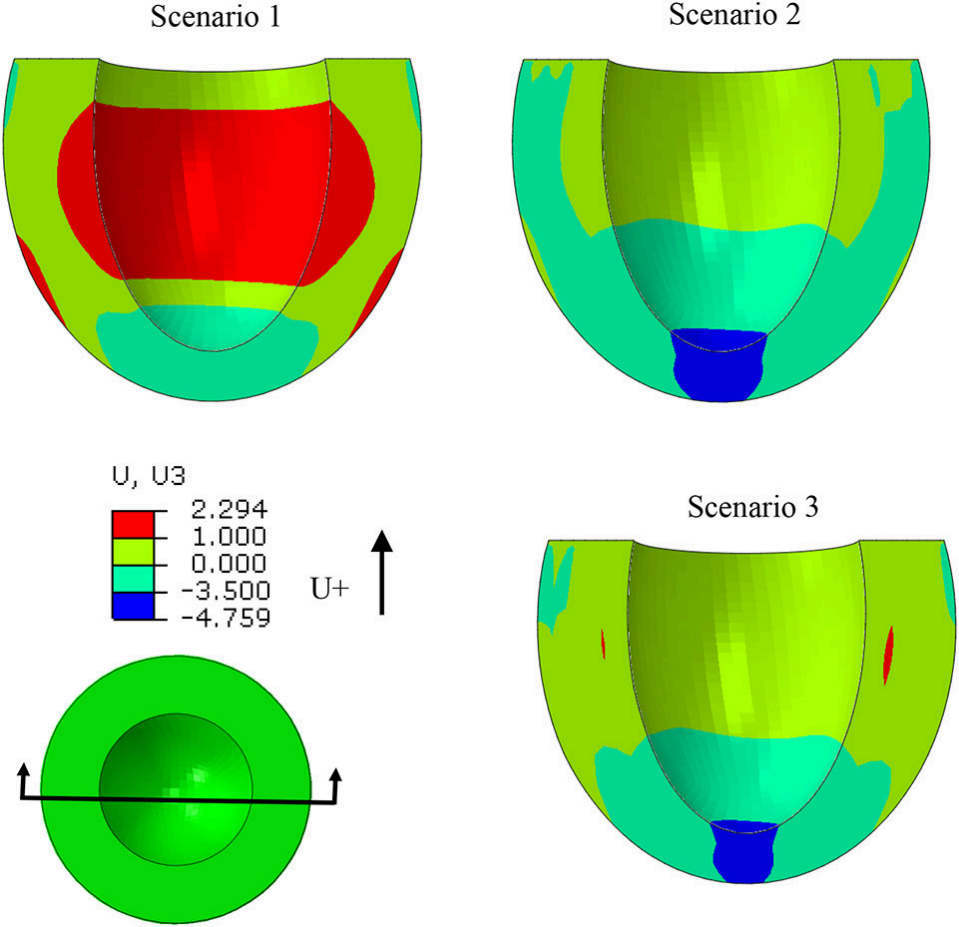
The ES longitudinal deformation was altered when subendocardial contractility was zero (scenarios 1 and 2). Deformation partially recovered when subepicardial contractility increased (scenarios 1 and 3).

The ES-shortening longitudinal displacement of the LV was profoundly decreased when subendocardial contractility was zero (scenarios 1 and 2, Figure 9). The longitudinal displacement was partially recovered when subepicardial contractility increased (scenarios 1 and 3, Figure 9).

The ES sphericity index (defined as the ratio between the lengths of the LV long axis and the short axis) was approximated as 1.1, 1.0, and 1.1 for scenarios 1, 2, and 3, respectively. In the HFrEF case (scenario 2), the ES sphericity index decreased compared to scenario 1. However, the ES sphericity index in the HFpEF scenario normalized toward the normal scenario. In other words, when the subendocardial contractility was zero, the LV shape became more spherical, compared to scenario 1. The shape of the LV recovered toward the normal scenario when subepicardial contractility increased.

## Discussion

In this study, we used a realistic FE model of the human LV to examine the role of altered LV systolic mechanics as a mechanism of HFpEF. Our findings support the hypothesis that HFpEF could be a result of lower subendocardial contractility linked with increased subepicardial contractility (Sengupta and Narula, 2008; Shah and Solomon, 2012; Omar et al., 2016, 2017). When subendocardial contractility was zero, LVEF decreased by 23.9% (Table 2: 53.2% in scenario 1 vs. 40.5% in scenario 2). The EF normalized when subepicardial contractility increased (Table 2: 53.2% in scenario 3 vs. 53.2% in scenario 1). The change in subepicardial contractility (less than a 40% increase from normal values) resulted in a 31.4% improvement in EF. Unlike scenario 1, scenarios 2 and 3 experienced abnormal strains within the subendocardial region (Figures 5–7), even though scenario 3 experienced normal EF. The ES sphericity index decreased in scenario 2 (1.0) compared to scenario 1 (1.1), but it recovered in scenario 3 (1.1). The LV torsion increased in scenario 2 (26.7°) compared to scenario 1 (24.7°), and it further increased in scenario 3 (30.2°).

The subendocardial region played an important role in the LV systolic mechanics, as our results showed. In particular, when subendocardial contractility was zero, the EF was reduced below 50%. A scenario with EF below 50% and zero subendocardial contractility corresponds to HFrEF (Vasan et al., 1999; Owan and Redfield, 2005; Yancy et al., 2013). Also, reducing EF below 50% by zeroing subendocardial contractility is in line with previous studies that reported the important role of the subendocardial region in the mechanics of the LV (Sabbah et al., 1981; Algranati et al., 2011). Based on our adopted definition of end-systolic elastance (E_ES_) (Chen et al., 2001), our results imply that E_ES_ decreased after subendocardial contractility was zero, but E_ES_ recovered when subepicardial contractility increased (Table 2, scenarios 2 and 3 vs. scenario 1). Increased ESP in HFpEF could be due to alterations in the ejection period of the LV (scenario 2). After subendocardial contractility was lost, the ejection period shortened and ended with a higher pressure.

The EF decreased by 75% when contractility decreased by 50% in all layers (scenario 4 vs. scenario 1, Table 2). On the other hand, setting subepicardial and midmyocardial contractility to zero affected EF more than subendocardial contractility (0.3% in scenario 5 and 12.7% in scenario 6 vs. 40.5% in scenario 2, Table 2). This result illustrates the important role of subepicardial and midmyocardial regions and confirms previous experimental (Haynes et al., 2014) and computational (Wang et al., 2016) studies that indicated the important roles of the epicardium and midmyocardium in systolic mechanics of the LV. Also, based on our adopted definition of E_ES_, this parameter decreased when contractility decreased in all layers by 50%, and when subepicardial and midmyocardial contractility were zero, and also when subepicardial contractility was set to zero (Table 2, scenarios 4, 5, and 6 vs. scenario 1).

Quantifying changes in torsional deformation related to changes in transmural contractility revealed an interesting relationship between the two. Abnormally high torsion could be a useful index of pathology, as we showed when subendocardial contractility was lost (Table 2, scenarios 2 and 3 vs. 1). This result confirms previous reports according to which the LV torsion increases in subendocardial ischemia (Prinzen et al., 1984), which has been related to the counter torque applied by the subendocardial region against the subepicardial region (Aelen et al., 1997). Also, this counter torque effect between subendocardium and subepicarium can be seen in scenarios 5 and 6 (Table 2). In these scenarios a negative torsion was seen after midmyocardium and subepicardium contractility was set to zero. The torsion of the LV is strongly coupled to the LV contractility and the inability to complete ejection properly (Table 2).

The longitudinal strain has been reported as a criterion to diagnose normal and diseased hearts (Henein and Gibson, 1999; Takeda et al., 2001; Yu et al., 2002; Vinereanu et al., 2005). The decreased longitudinal strain in our results (Table 2) corresponds to clinical studies that reported longitudinal strains decrease in HFpEF (Mizuguchi et al., 2010). Also, the contour of ES longitudinal displacement, which is directly related to longitudinal strain, was noticeably altered in the diseased scenario compared to the normal scenario (Figure 9, scenarios 1 and 2). However, when subepicardial contractility increased, the pattern of longitudinal displacement became more similar to the normal scenario (Figure 9, scenarios 1 and 3). The ES strain pattern across the regional layers of the LV wall (Figure 7) also supported the hypothesis that increased subepicardial contractility in HFpEF improves function globally (Sengupta and Narula, 2008). Moreover, the alterations in circumferential and radial strains are in line with clinical studies that reported these strains decrease in HFpEF (Wang et al., 2008; Mizuguchi et al., 2010). Yet, our results should be interpreted with caution. The circumferential and radial strains for normal conditions (scenario 1) were in line with clinical data reported in the literature, whereas the longitudinal strain was smaller than reported clinical data (Moore et al., 2000; Yingchoncharoen et al., 2013). The methodology of our study is similar to previous computational models of LV in our group. The longitudinal strain results of these previous models have been validated against experimental strain data (for example, Genet et al., 2014).

It has been well-documented that the shape of the LV changes in HF (Grossman et al., 1975; Carabello, 1995; Gaasch and Zile, 2011). In particular, LV concentric hypertrophy is seen in patients with HFpEF (Melenovsky et al., 2007), and exercise capacity is correlated with the sphericity index of the LV (Tischler et al., 1993). In line with previous studies, in our simulations, the shape of the LV was altered when the subendocardial contractility was zero. The ES sphericity index decreased in scenario 2 (1.0) compared to scenario 1 (1.1). When subepicardial contractility increased, the shape of the LV recovered toward the normal case, as seen in the ES sphericity index in scenario 3 (1.1) compared to scenario 2 (1.0). Thus, the increased subepicardial contractility may prevent LV dilatation in HFpEF, and help preserve the LV shape. This phenomenon will further support the normalization of LVEF due to the direct interplay between the LV shape and function (Grossman et al., 1975; Stokke et al., 2017).

In this study we used tissue-level load-independent properties (T_max_) to alter myocardium contractility. This approach is more appropriate than using the LV strains. In fact, the popular notion of equating myocardial contractility with strain measurements (that are load dependent) is “off the mark [and] if contractility means anything, it is as an expression of the ability of a given piece of myocardium to generate tension and shortening under any loading conditions” (Reichek, 2013). Therefore, our approach to alter transmural contractility, which might not be feasible using current experimental methods, could lead to a better understanding of the development of HFpEF.

Novel physics-based mathematical modeling was used in this study to examine a possible mechanism underlying preservation of LVEF in HFpEF. The results from the simulations provide evidence of the potential role of myocardial contractility in the genesis of preserved EF in the HFpEF phenotype. Previous studies on HFpEF were mostly based on experimental data (for example, Phan et al., 2009), where the contribution of a single feature like myocardial contractility could not be varied in isolation of other parameters. However, we used FE modeling to simulate and isolate transmural contractility as a feature and study its effect on LV systolic mechanics. Our results provide important first steps toward eventual development of a computational model of HFpEF.

### Study limitations and future directions

The simulation addressed only the relationship between transmural myocardial contractility and LV systolic mechanics. Modeling all aspects of HFpEF was beyond the scope of this paper. In clinical conditions, several factors contribute to the development of HFpEF (Bench et al., 2009; Shah and Solomon, 2012; Sengupta and Marwick, 2018). These factors include abnormalities in both the systolic and the diastolic mechanics of the LV (MacIver and Townsend, 2008; MacIver, 2009; Shah and Solomon, 2012), the LV hypertrophy and geometric changes (Aurigemma et al., 1995; Vasan et al., 1999; Adeniran et al., 2015) and material properties of the LV (stiffness). A recent study employed computational models with heterogeneous transmural distributions of T_max_ (Wang et al., 2016). In our investigation, only in one scenario (scenario 1) did we assume T_max_ to be uniform in the transmural direction. Also, since this study focused on the LV, we assumed timing and activation of contractility are homogenous. A more realistic assumption would be to consider the sequence of electrical stimulations in the tissue (Chabiniok et al., 2012; Villongco et al., 2014; Crozier et al., 2016; Giffard-Roisin et al., 2017), particularly in the septum. However, a heterogeneous distribution of T_max_ would be more important if atria were also included in the model. Moreover, we only modeled LV data from one human subject in our study. Modeling data from multiple subjects is the goal of a subsequent study. Here, our intent was to document our modeling methodology and demonstrate its utility.

Alterations in strain distributions might lead to remodeling in the LV tissue (Figures 5–7). The response of myocardial tissue to an altered mechanical environment will likely lead to changes in tissue properties that will in turn affect the LV inflation, contraction, and relaxation. It is well-documented that diastolic LV tissue stiffness becomes abnormally high in HFpEF (Zile et al., 2004). Our study focused on the systolic mechanics of the LV. As a future direction, integration of tissue response in diastole and systole will provide a more realistic and informative model to understand the mechanisms involved during the onset and development of HFpEF. Integration of cell-based cross-bridge cycling and contractility could provide more realistic information about the tissue alterations over the course of HFpEF development (Adeniran et al., 2015; Shavik et al., 2017).

Although our study explored a simplified representation of HFpEF (appropriately so, to isolate mechanical effects), the clinical definition and diagnosis of HFpEF and HFrEF are more complex than just calculations of EF (Borlaug and Paulus, 2011). In fact, HFpEF lacks a clear validated diagnostic guideline (Lam, 2010; Oghlakian et al., 2011). In this hypothesis-generating study, we simply assumed EF < 50% represents HFrEF. This assumption is in line with some definitions used for HFrEF in the literature (Vasan et al., 1999; Paulus et al., 2007). However, an EF = 40.5% (scenario 2) might also be defined as borderline HFpEF (for example, Yancy et al., 2013). These points may be considered semantic because they do not affect the conclusions of our study, which quantified alterations in contractility with changes in EF, torsion and strain. It would be interesting to apply these methods to personalized models derived from patients diagnosed clinically with HFpEF and HFrEF.

Several other scenarios need to be investigated, including more graded loss of subendocardial contractility, and graded decrease of subendocardial contractility, with both coupled to a graded increase in subepicardial contractility. Moreover, the definitions of subendocardium, midmyocardium, and subepicardium regions were arbitrary in this study because exact definitions are not available. A more realistic imaging approach might better delineate transmural layers and their related contractility. Furthermore, exercise intolerance has been reported as a key factor in HFpEF (Roh et al., 2017), and could be implemented in our modeling methodology to better understand the mechanisms of HFpEF development. Despite these limitations, this paper reports instructive quantitative information about development of HFpEF, as we could change one aspect of the model (contractility at a particular location) and determine its effects alone.

## Conclusions

The results of this study support the hypothesis that preservation of LVEF in patients with HFpEF could be explained on the basis of reduced subendocardial contractility with a compensatory increase in subepicardial contractility. These findings underscore the roles of regional LV myocardial contractility in HF syndromes and emphasize the importance of computational models in understanding pathophysiological mechanisms underlying complex phenotypic presentations like HFpEF.

## Author contributions

PS and JG designed the study. KS developed the constitutive model. YD and KS created the computational models. YD ran simulations, compiled results, and wrote the initial draft of the paper. SS helped with the modeling process. YD, KS, JG, and PS contributed to analysis of the results, and manuscript writing.

### Conflict of interest statement

The authors declare that the research was conducted in the absence of any commercial or financial relationships that could be construed as a potential conflict of interest.

## References

[B1] Adeniran I. MacIver D. H. Hancox J. C. Zhang H. (2015). Abnormal calcium homeostasis in heart failure with preserved ejection fraction is related to both reduced contractile function and incomplete relaxation: an electromechanically detailed biophysical modeling study. Front. Physiol. 6:78. 10.3389/fphys.2015.00078 25852567 PMC4367530

[B2] Aelen F. W. Arts T. Sanders D. G. Thelissen G. R. Muijtjens A. M. Prinzen F. W.. (1997). Relation between torsion and cross-sectional area change in the human left ventricle. J. Biomech. 30, 207–212. 10.1016/S0021-9290(96)00147-9 9119819

[B3] Algranati D. Kassab G. S. Lanir Y. (2011). Why is the subendocardium more vulnerable to ischemia? A new paradigm. Am. J. Physiol. Heart Circ. Physiol. 300, H1090–H1100. 10.1152/ajpheart.00473.2010 21169398 PMC3064294

[B4] Aurigemma G. P. Gaasch W. H. (2004). Clinical practice. Diastolic heart failure. N. Engl. J. Med. 351, 1097–1105. 10.1056/NEJMcp022709 15356307

[B5] Aurigemma G. P. Silver K. H. Priest M. A. Gaasch W. H. (1995). Geometric changes allow normal ejection fraction despite depressed myocardial shortening in hypertensive left ventricular hypertrophy. J. Am. Coll. Cardiol. 26, 195–202. 10.1016/0735-1097(95)00153-Q 7797752

[B6] Baillargeon B. Costa I. Leach J. R. Lee L. C. Genet M. Toutain A.. (2015). Human cardiac function simulator for the optimal design of a novel annuloplasty ring with a sub-valvular element for correction of ischemic mitral regurgitation. Cardiovasc. Eng. Technol. 6, 105–116. 10.1007/s13239-015-0216-z 25984248 PMC4427655

[B7] Baillargeon B. Rebelo N. Fox D. D. Taylor R. L. Kuhl E. (2014). The living heart project: a robust and integrative simulator for human heart function. Eur. J. Mech. A Solids 48, 38–47. 10.1016/j.euromechsol.2014.04.001 25267880 PMC4175454

[B8] Bench T. Burkhoff D. O'Connell J. B. Costanzo M. R. Abraham W. T. St John Sutton M.. (2009). Heart failure with normal ejection fraction: consideration of mechanisms other than diastolic dysfunction. Curr. Heart Fail. Rep. 6, 57–64. 10.1007/s11897-009-0010-z 19265594

[B9] Benjamin E. J. Blaha M. J. Chiuve S. E. Cushman M. Das S. R. Deo R. (2017). American heart association statistics committee and stroke statistics Subcommittee. heart disease and stroke statistics-2017 update: a report from the American Heart Association. Circulation 135, e146–e603. 10.1161/CIR.0000000000000485 28122885 PMC5408160

[B10] Bhuiyan T. Maurer M. S. (2011). Heart failure with preserved ejection fraction: persistent diagnosis, therapeutic enigma. Curr. Cardiovasc. Risk Rep. 5, 440–449. 10.1007/s12170-011-0184-2 22081782 PMC3211140

[B11] Borlaug B. A. Paulus W. J. (2011). Heart failure with preserved ejection fraction: pathophysiology, diagnosis, and treatment. Eur. Heart J. 32, 670–679. 10.1093/eurheartj/ehq426 21138935 PMC3056204

[B12] Carabello B. A. (1995). The relationship of left ventricular geometry and hypertrophy to left ventricular function in valvular heart disease. J. Heart Valve Dis. 4(Suppl. 2), S132–S138. 8563989

[B13] Carrick R. Ge L. Lee L. C. Zhang Z. Mishra R. Axel L.. (2012). Patient-specific finite element-based analysis of ventricular myofiber stress after Coapsys: importance of residual stress. Ann. Thorac. Surg. 93, 1964–1971. 10.1016/j.athoracsur.2012.03.001 22560323 PMC3470864

[B14] Chabiniok R. Moireau P. Lesault P. F. Rahmouni A. Deux J. F. Chapelle D. (2012). Estimation of tissue contractility from cardiac cine-MRI using a biomechanical heart model. Biomech. Model. Mechanobiol. 11, 609–630. 10.1007/s10237-011-0337-8 21796413

[B15] Chen C. H. Fetics B. Nevo E. Rochitte C. E. Chiou K. R. Ding P. A.. (2001). Noninvasive single-beat determination of left ventricular end-systolic elastance in humans. J. Am. Coll. Cardiol. 38, 2028–2034. 10.1016/S0735-1097(01)01651-5 11738311

[B16] Crozier A. Blazevic B. Lamata P. Plank G. Ginks M. Duckett S. (2016). The relative role of patient physiology and device optimisation in cardiac resynchronisation therapy: a computational modelling study. J. Mol. Cell. Cardiol. 96, 93–100. 10.1016/j.yjmcc.2015.10.026 26546827 PMC4915816

[B17] Gaasch W. H. Zile M. R. (2011). Left ventricular structural remodeling in health and disease: with special emphasis on volume, mass, and geometry. J. Am. Coll. Cardiol. 58, 1733–1740. 10.1016/j.jacc.2011.07.022 21996383

[B18] Genet M. Lee L. C. Nguyen R. Haraldsson H. Acevedo-Bolton G. Zhang Z. (2014). Distribution of normal human LV myofiber stress at end diastole and end systole: a target for *in silico* design of heart failure treatments. J. Appl. Physiol. 117, 142–152. 10.1152/japplphysiol.00255.2014 24876359 PMC4101610

[B19] Giffard-Roisin S. Jackson Th Fovargue L. Lee J. Delingette H. Razavi R.. (2017). Noninvasive personalization of a cardiac electrophysiology model from body surface potential mapping. IEEE Trans. Biomed. Eng. 64, 2206–2218. 10.1109/TBME.2016.2629849 28113292

[B20] Göktepe S. Acharya S. N. S. Wong J. Kuhl E. (2011). Computational modeling of passive myocardium. Int. J. Numer. Methods Biomed. Eng. 27, 1–12. 10.1002/cnm.1402

[B21] Grossman W. Jones D. McLaurin L. P. (1975). Wall stress and patterns of hypertrophy in the human left ventricle. J. Clin. Invest. 56, 56–64. 10.1172/JCI108079 124746 PMC436555

[B22] Guccione J. M. McCulloch A. D. (1993). Mechanics of active contraction in cardiac muscle. I. Constitutive relations for fiber stress that describe deactivation. J. Biomech. Eng. 115, 72–81. 10.1115/1.2895473 8445901

[B23] Haynes P. Nava K. E. Lawson B. A. Chung C. S. Mitov M. I. Campbell S. G.. (2014). Transmural heterogeneity of cellular level power output is reduced in human heart failure. J. Mol. Cell Cardiol. 72, 1–8. 10.1016/j.yjmcc.2014.02.008 24560668 PMC4037376

[B24] Heidenreich P. A. Albert N. M. Allen L. A. Bluemke D. A. Butler J. Fonarow G. C.. (2013). Forecasting the impact of heart failure in the United States: a policy statement from the American Heart Association. Circ. Heart Fail. 6, 606–619. 10.1161/HHF.0b013e318291329a 23616602 PMC3908895

[B25] Henein M. Y. Gibson D. G. (1999). Long axis function in disease. Heart 81, 229–231. 10.1136/hrt.81.3.229 10026340 PMC1728969

[B26] Holzapfel G. A. Ogden R. W. (2009). Constitutive modelling of passive myocardium: a structurally based framework for material characterization. Philos. Trans. A Math. Phys. Eng. Sci. 367, 3445–3475. 10.1098/rsta.2009.0091 19657007

[B27] Jones E. Oliphant T. Peterson P. (2001). SciPy: Open Source Scientific Tools for Python. Available online at: http://www.scipy.org

[B28] Klotz S. Hay I. Dickstein M. L. Yi G. H. Wang J. Maurer M. S.. (2006). Single-beat estimation of end-diastolic pressure-volume relationship: a novel method with potential for noninvasive application. Am. J. Physiol. Heart Circ. Physiol. 291, H403–H412. 10.1152/ajpheart.01240.2005 16428349

[B29] Lam C. S. (2010). Heart failure with preserved ejection fraction: invasive solution to diagnostic confusion? J. Am. Coll. Cardiol. 55, 1711–1712. 10.1016/j.jacc.2009.12.034 20394875

[B30] Lee L. C. Genet M. Acevedo-Bolton G. Ordovas K. Guccione J. M. Kuhl E. (2015). A computational model that predicts reverse growth in response to mechanical unloading. Biomech. Model. Mechanobiol. 14, 217–229. 10.1007/s10237-014-0598-0 24888270 PMC4254895

[B31] Lee L. C. Zhihong Z. Hinson A. Guccione J. M. (2013). Reduction in left ventricular wall stress and improvement in function in failing hearts using Algisyl-LVR. J. Vis. Exp. 74:50096 10.3791/50096 PMC3653384 23608998

[B32] LeGrice I. Hunter P. Young A. Small B. (2001). The architecture of the heart: a data-based model. Philos. Trans. R. Soc. Lond. Ser. A Math. Phys. Eng. Sci. 359, 1217–1232. 10.1098/rsta.2001.0827

[B33] Lombaert H. Peyrat J.-M. Croisille P. Rapacchi S. Fanton L. Clarysse P.. (2011). Statistical analysis of the human cardiac fiber architecture from DT-MRI, in Functional Imaging and Modeling of the Heart (Berlin; Heidelberg: Springer), 171–179.

[B34] MacIver D. H. (2009). Heart failure with preserved ejection fraction: is it due to contractile dysfunction? Circ. J. 73:1169. 10.1253/circj.CJ-09-0190 19465785

[B35] MacIver D. H. Townsend M. (2008). A novel mechanism of heart failure with normal ejection fraction. Heart 94, 446–449. 10.1136/hrt.2006.114082 17483129

[B36] Melenovsky V. Borlaug B. A. Rosen B. Hay I. Ferruci L. Morell C. H.. (2007). Cardiovascular features of heart failure with preserved ejection fraction versus nonfailing hypertensive left ventricular hypertrophy in the urban Baltimore community: the role of atrial remodeling/dysfunction. J. Am. Coll. Cardiol. 49, 198–207. 10.1016/j.jacc.2006.08.050 17222731

[B37] Mercier J. C. DiSessa T. G. Jarmakani J. M. Nakanishi T. Hiraishi S. Isabel-Jones J.. (1982). Two-dimensional echocardiographic assessment of left ventricular volumes and ejection fraction in children. Circulation 65, 962–969. 10.1161/01.CIR.65.5.962 7074761

[B38] Mizuguchi Y. Oishi Y. Miyoshi H. Iuchi A. Nagase N. Oki T. (2010). Concentric left ventricular hypertrophy brings deterioration of systolic longitudinal, circumferential, and radial myocardial deformation in hypertensive patients with preserved left ventricular pump function. J. Cardiol. 55, 23–33. 10.1016/j.jjcc.2009.07.006 20122545

[B39] Moore C. C. Lugo-Olivieri C. H. McVeigh E. R. Zerhouni E. A. (2000). Three-dimensional systolic strain patterns in the normal human left ventricle: characterization with tagged MR imaging. Radiology 214, 453–466. 10.1148/radiology.214.2.r00fe17453 10671594 PMC2396279

[B40] Nagueh S. F. Middleton K. J. Kopelen H. A. Zoghbi W. A. Quiñones M. A. (1997). Doppler tissue imaging: a noninvasive technique for evaluation of left ventricular relaxation and estimation of filling pressures. J. Am. Coll. Cardiol. 30, 1527–1533. 10.1016/S0735-1097(97)00344-6 9362412

[B41] Oghlakian G. O. Sipahi I. Fang J. C. (2011). Treatment of heart failure with preserved ejection fraction: have we been pursuing the wrong paradigm? Mayo Clin. Proc. 86, 531–539. 21576513 10.4065/mcp.2010.0841 PMC3104912

[B42] Omar A. M. Bansal M. Sengupta P. P. (2016). Advances in echocardiographic imaging in heart failure with reduced and preserved ejection fraction. Circ. Res. 119, 357–374. 10.1161/CIRCRESAHA.116.309128 27390337

[B43] Omar A. M. S. Narula S. Abdel Rahman M. A. Pedrizzetti G. Raslan H. Rifaie O.. (2017). Precision phenotyping in heart failure and pattern clustering of ultrasound data for the assessment of diastolic dysfunction. JACC Cardiovasc. Imaging 10, 1291–1303. 10.1016/j.jcmg.2016.10.012 28109936

[B44] Owan T. E. Redfield M. M. (2005). Epidemiology of diastolic heart failure. Prog. Cardiovasc. Dis. 47, 320–332. 16003647 10.1016/j.pcad.2005.02.010

[B45] Paulus W. J. Tschöpe C. Sanderson J. E. Rusconi C. Flachskampf F. A. (2007). How to diagnose diastolic heart failure: a consensus statement on the diagnosis of heart failure with normal left ventricular ejection fraction by the Heart Failure and Echocardiography Associations of the European Society of Cardiology. Eur. Heart J. 28, 2539–2550. 10.1093/eurheartj/ehm037 17428822

[B46] Phan T. T. Abozguia K. Nallur Shivu G. Mahadevan G. Ahmed I. Williams L.. (2009). Heart failure with preserved ejection fraction is characterized by dynamic impairment of active relaxation and contraction of the left ventricle on exercise and associated with myocardial energy deficiency. J. Am. Coll. Cardiol. 54, 402–409. 10.1016/j.jacc.2009.05.012 19628114

[B47] Prinzen F. W. Arts T. van der Vusse G. J. Reneman R. S. (1984). Fiber shortening in the inner layers of the left ventricular wall as assessed from epicardial deformation during normoxia and ischemia. J. Biomech. 17, 801–811. 6526839 10.1016/0021-9290(84)90111-8

[B48] Rüssel I. K. Götte M. J. Bronzwaer J. G. Knaapen P. Paulus W. J. van Rossum A. C. (2009). Left ventricular torsion: an expanding role in the analysis of myocardial dysfunction. JACC Cardiovasc. Imaging 2, 648–655. 10.1016/j.jcmg.2009.03.001 19442954

[B49] Reichek N. (2013). Right ventricular strain in pulmonary hypertension: flavor du jour or enduring prognostic index? Circul. Cardiovasc. Imaging 6, 609–611. 10.1161/CIRCIMAGING.113.000936 24046376

[B50] Roh J. Houstis N. Rosenzweig A. (2017). Why Don't we have proven treatments for HFpEF? Circ. Res. 120, 1243–1245. 10.1161/CIRCRESAHA.116.310119 28408453 PMC5407384

[B51] Sabbah H. N. Marzilli M. Stein P. D. (1981). The relative role of subendocardium and subepicardium in left ventricular mechanics. Am. J. Physiol. 240, H920–H926. 7246754 10.1152/ajpheart.1981.240.6.H920

[B52] Sack K. L. Baillargeon B. Acevedo-Bolton G. Genet M. Rebelo N. Kuhl E. (2016). Partial LVAD restores ventricular outputs and normalizes LV but not RV stress distributions in the acutely failing heart in silico. Int. J. Artif. Organs. 39, 421–430. 10.5301/ijao.5000520 27646633 PMC5067236

[B53] Sengupta P. P. Marwick T. H. (2018). The many dimensions of diastolic function: a curse or a blessing? JACC Cardiovasc. Imaging. 11, 409–410. 10.1016/j.jcmg.2017.05.015 28734924

[B54] Sengupta P. P. Narula J. (2008). Reclassifying heart failure: predominantly subendocardial, subepicardial, and transmural. Heart Fail. Clin. 4, 379–382. 10.1016/j.hfc.2008.03.013 18598989

[B55] Shah A. M. Solomon S. D. (2012). Phenotypic and pathophysiological heterogeneity in heart failure with preserved ejection fraction. Eur. Heart J. 33, 1716–1717. 10.1093/eurheartj/ehs124 22730487

[B56] Shavik S. M. Wall S. T. Sundnes J. Burkhoff D. Lee L. C. (2017). Organ-level validation of a cross-bridge cycling descriptor in a left ventricular finite element model: effects of ventricular loading on myocardial strains. Physiol. Rep. 5:e13392. 10.14814/phy2.13392 29122952 PMC5688770

[B57] Steinberg B. A. Zhao X. Heidenreich P. A. Peterson E. D. Bhatt D. L. Cannon C. P.. (2012). Trends in patients hospitalized with heart failure and preserved left ventricular ejection fraction: prevalence, therapies, and outcomes. Circulation 126, 65–75. 10.1161/CIRCULATIONAHA.111.080770 22615345

[B58] Stokke T. M. Hasselberg N. E. Smedsrud M. K. Sarvari S. I. Haugaa K. H. Smiseth O. A.. (2017). Geometry as a confounder when assessing ventricular systolic function: comparison between ejection fraction and strain. J. Am. Coll. Cardiol. 70, 942–954. 10.1016/j.jacc.2017.06.046 28818204

[B59] Streeter D. D. Spotnitz H. M. Patel D. P. Ross J. Sonnenblick E. H. (1969). Fiber orientation in the canine left ventricle during diastole and systole. Circ. Res. 24, 339–347. 5766515 10.1161/01.res.24.3.339

[B60] Takeda S. Rimington H. Smeeton N. Chambers J. (2001). Long axis excursion in aortic stenosis. Heart 86, 52–56. 10.1136/heart.86.1.52 11410562 PMC1729828

[B61] Tischler M. D. Niggel J. Borowski D. T. LeWinter M. M. (1993). Relation between left ventricular shape and exercise capacity in patients with left ventricular dysfunction. J. Am. Coll. Cardiol. 22, 751–757. 8354809 10.1016/0735-1097(93)90187-6

[B62] Vasan R. S. Benjamin E. J. Levy D. (1995). Prevalence, clinical features and prognosis of diastolic heart failure: an epidemiologic perspective. J. Am. Coll. Cardiol. 26, 1565–1574. 7594087 10.1016/0735-1097(95)00381-9

[B63] Vasan R. S. Larson M. G. Benjamin E. J. Evans J. C. Reiss C. K. Levy D. (1999). Congestive heart failure in subjects with normal versus reduced left ventricular ejection fraction: prevalence and mortality in a population-based cohort. J. Am. Coll. Cardiol. 33, 1948–1955. 10362198 10.1016/s0735-1097(99)00118-7

[B64] Villongco C. T. Krummen D. E. Stark P. Omens J. H. McCulloch A. D. (2014). Patient-specific modeling of ventricular activation pattern using surface ECG-derived vectorcardiogram in bundle branch block. Prog. Biophys. Mol. Biol. 115, 305–313. 10.1016/j.pbiomolbio.2014.06.011 25110279 PMC4254140

[B65] Vinereanu D. Nicolaides E. Tweddel A. C. Fraser A. G. (2005). “Pure” diastolic dysfunction is associated with long-axis systolic dysfunction. Implications for the diagnosis and classification of heart failure. Eur. J. Heart Fail. 7, 820–828. 10.1016/j.ejheart.2005.02.003 15921957

[B66] Walker J. C. Ratcliffe M. B. Zhang P. Wallace A. W. Fata B. Hsu E. W.. (2005). MRI-based finite-element analysis of left ventricular aneurysm. Am. J. Physiol. Heart Circ. Physiol. 289, H692–H700. 10.1152/ajpheart.01226.2004 15778283

[B67] Wang H. Zhang X. Dorsey S. M. McGarvey J. R. Campbell K. S. Burdick J. A.. (2016). Computational investigation of transmural differences in left ventricular contractility. ASME J. Biomech. Eng. 138:114501. 10.1115/1.4034558 27591094 PMC5125313

[B68] Wang J. Khoury D. S. Yue Y. Torre-Amione G. Nagueh S. F. (2008). Preserved left ventricular twist and circumferential deformation, but depressed longitudinal and radial deformation in patients with diastolic heart failure. Eur. Heart J. 29, 1283–1289. 10.1093/eurheartj/ehn141 18385117

[B69] Yancy C. W. Jessup M. Bozkurt B. Butler J. Casey D. E. Jr Drazner M. H.. (2013). ACCF/AHA guideline for the management of heart failure: a report of the American College of Cardiology Foundation/American Heart Association task force on practice guidelines. J. Am. Coll. Cardiol. 62, e147–e239. 10.1016/j.jacc.2013.05.019 23747642

[B70] Yingchoncharoen T. Agarwal S. Popović Z. B. Marwick T. H. (2013). Normal ranges of left ventricular strain: a meta-analysis. J. Am. Soc. Echocardiogr. 26, 185–191. 10.1016/j.echo.2012.10.008 23218891

[B71] Yu C. M. Lin H. Yang H. Kong S. L. Zhang Q. Lee S. W. (2002). Progression of systolic abnormalities in patients with “isolated” diastolic heart failure and diastolic dysfunction. Circulation. 105, 1195–1201. 10.1161/hc1002.105185 11889013

[B72] Zile M. R. Baicu C. F. Gaasch W. H. (2004). Diastolic heart failure–abnormalities in active relaxation and passive stiffness of the left ventricle. N. Engl. J. Med. 350, 1953–1959. 10.1056/NEJMoa032566 15128895

